# Efficacy of Selective Laser Trabeculoplasty after iStent Implantation in Primary Open-Angle Glaucoma

**DOI:** 10.3390/jpm11080797

**Published:** 2021-08-16

**Authors:** Adam R Siedlecki, Patrice M Hicks, Benjamin Haaland, Margaret M DeAngelis, Sandra F Sieminski

**Affiliations:** 1 Department of Ophthalmology, Ross Eye Institute, Jacobs School of Medicine and Biomedical Engineering, SUNY-University at Buffalo, Buffalo, NY 14214, USA; as222@buffalo.edu (A.R.S.); mmdeange@buffalo.edu (M.M.D.); 2 Department of Population Health Sciences, University of Utah School of Medicine, Salt Lake City, UT 84108, USA; patrice.hicks@hsc.utah.edu (P.M.H.); ben.haaland@hsc.utah.edu (B.H.); 3 Department of Ophthalmology and Visual Sciences, University of Utah School of Medicine, Salt Lake City, UT 84132, USA; 4 VA Western New York Healthcare System, Buffalo, NY 14215, USA

**Keywords:** glaucoma, iStent, SLT

## Abstract

iStent implantation is thought to augment the trabecular outflow channel in the anterior segment of the eye. We hypothesized that iStent with subsequent selective laser trabeculoplasty (SLT) would better control the intraocular pressure (IOP) compared to standalone SLT in patients with primary open-angle glaucoma (POAG). We, therefore, determined if the presence of an iStent combined with SLT was statistically associated with IOP lowering compared to standalone SLT. Through retrospective electronic medical record review, records of 824 eyes from 440 patients who received primary SLT without a history of iStent were considered. Additionally, 42 eyes from 28 patients who received SLT after combined phacoemulsification and iStent implantation that failed to control intraocular pressure (IOP) and/or the progression of the disease were retrospectively reviewed. IOP and number of medications, which were tracked in each patient for up to 12 months post laser, were also examined. Successful outcome was defined as a statistically significant reduction in IOP or number of medications at 6 months. As defined in univariate analysis *(p* ≤ 0.01), multivariate analysis included iStent, age, sex, race, and initial IOP as variables. IOP reduction was statistically associated with patients pre-SLT IOP (*p* < 0.001) but not with patients with iStent (*p* = 0.222). Medication reduction was statistically associated with the pre-SLT number of medications (*p* < 0.001) and iStent (*p* < 0.001). In eyes that received SLT, iStent was not statistically associated with a greater reduction in IOP compared to controls, but was associated with a higher reduction in the overall number of medications used 6 months after receiving SLT. The work presented should guide clinicians to consider SLT as an effective therapy after iStent implantation, in terms of glaucoma medication reduction in iStent patients, but clinicians should know that the presence of an iStent does not necessarily make subsequent SLT more effective at lowering IOP.

## 1. Introduction

Glaucoma is a heterogeneous group of eye diseases associated with intraocular pressure and optic nerve damage, resulting in characteristic visual field loss patterns and eventual blindness if untreated [1]. It is the leading cause of blindness worldwide [2]. Glaucoma is subdivided into primary versus secondary causes and open versus closed angles. Primary open-angle glaucoma is a chronic, progressive form of glaucoma characterized by gradual loss of retinal ganglion cells and optic nerve atrophy [3]. Increased IOP is a significant risk factor associated with the development and progression of glaucomatous damage to the optic nerve, resulting in blindness [3,4]. Thus, controlling IOP is a mainstay of clinical and surgical management for glaucoma patients, even for patients whose IOP is in the normal range [5].

Presently, glaucoma is managed along a ladder of treatment options ranging from least-invasive to most-invasive [6]. Although many patients are successfully treated with medical therapy in the form of eye drops to lower IOP, some patients require laser or incisional surgical intervention. Many of these surgeries lower IOP by augmenting the trabecular, or conventional, outflow pathway, while others bypass this outflow pathway altogether by establishing a new outflow route for aqueous to drain out of the anterior chamber. 

Laser treatments such as argon laser trabeculoplasty (ALT) and SLT are therapies that can help decrease medication burden, allow additional IOP control while continuing topical medication, or control IOP in the face of medication adherence issues. SLT involves a 532 nm frequency-doubled, Q-switched Nd: YAG laser with a three nanosecond pulse duration [7,8,9]. It was introduced in 1995 by Latina and Park as an evolution of ALT but with reduced energy delivered to the trabecular meshwork (TM), causing less thermal injury and scarring but with comparable effects on increasing aqueous outflow through the TM and reducing IOP. The mechanism by which SLT improves TM outflow is based on the upregulation of specific genes, cytokines, metalloproteinases, and TM remodeling [10,11,12,13,14,15]. The safety profile and lack of TM scarring in SLT allow for patients to receive SLT again in the future; additionally, repeated SLT procedures might achieve similar success in IOP control as seen in primary SLT [16,17].

The first-generation iStent (Glaukos Corporation, Laguna Hills, CA, USA) is a trabecular meshwork bypass device approved by the FDA in 2012 in conjunction with cataract surgery to lower IOP by increasing outflow through the TM [6,18]. The iStent is a heparin-coated, non-ferromagnetic titanium stent that is implanted into the TM using an ab interno approach. Multiple studies have demonstrated the effectiveness of iStent at lowering IOP and medication reduction in patients undergoing cataract surgery [19,20,21,22,23,24,25,26].

The purpose of this study is to investigate the efficacy of SLT after combined phacoemulsification and iStent implantation failed to control the IOP and/or progression of glaucoma. Secondarily, the study aims to investigate if SLT augments the increased trabecular outflow that may occur after iStent implantation and is, therefore, more effective than standalone SLT. Prior studies in this field have examined the effectiveness of SLT after prior failed ALT, primary trabeculectomy, phacoemulsification cataract extraction combined with ab interno trabeculotomy, and canaloplasty [27,28,29,30,31]. Current literature in this field has not yet investigated the use of SLT in patients who have undergone TM bypass shunts such as the iStent.

## 2. Materials and Methods

### 2.1. Data Collection

Data were retrospectively collected from patient charts at the Ross Eye Institute in Buffalo, New York. Billing data were used to generate a list of patients who had received SLT in the last five years and patients who had received iStent in the last five years. We obtained records of 824 eyes from a total of 440 patients who received primary SLT without a history of iStent, and 42 eyes from 28 patients who received SLT after failed iStent were ascertained and retrospectively reviewed. The decision to perform SLT in the selected patients was made based on the patients’ IOP, a progression of glaucomatous changes on Humphrey visual fields (HVF) test, the number of medications, and difficulties adhering to regular medication usage. This study received institutional review board approval from the Institutional Review Board at the University at Buffalo. This study conformed to the tenets of the Declaration of Helsinki. Study participants were enrolled in this study after giving written informed consent.

The following variables were collected from patients in the experimental group: age, gender, race, Hispanic ethnicity, glaucoma diagnosis grade (determined by visual field results before iStent or SLT interventions), other ocular histories, smoking status, diabetic status, essential hypertension status, hyperlipidemia status, other listed medical history, prior laser surgeries the patient received (in either eye), OS or OD, pachymetry; pre-iStent IOP, visual acuity (VA), number of medications; post-iStent IOP, VA, and number of medications at 1 day, 1 week, 1 month, 3 months, 5 to 8 months, 9 months, 12 months, 20 to 24 months, and 25 to 33 months; pre-SLT IOP, VA, and number of medications; and post-SLT IOP, VA, and number of medications at 2 to 6 weeks, 3 to 5 months, 5 to 8 months, and 9 to 12 months. Combination drops such as fixed combination dorzolamide 2%-timolol 0.5% were counted as two medications to account for both pharmacologic components. Latanoprostene bunod 0.024%, despite containing two molecular compounds, was counted as one medication. The same variables were collected from patients in the control group as previously described above with two exceptions: no iStent data was collected, but self-reported medication adherence data was collected. Medication adherence was ascertained by asking the patient, “What drops are you using, and how often do you take them?”

### 2.2. Inclusion and Exclusion Criteria for Data Analysis

The experimental group’s inclusion criteria were adults over 18 years old with no upper limit, a diagnosis of POAG, and prior failed iStent implanted in the last 5 years. Failed iStent was defined as a rise in IOP by 10% after 24 months or progression of glaucoma based on VF regardless of IOP change.

Inclusion criteria for the control group were eyes from patients over 18 years old with no upper limit and receiving SLT in the last 5 years. Exclusion criteria for the control group were eyes with non-POAG diagnoses, prior history of SLT, eyes of patients whose other eye received SLT (in the case of a patient who had both eyes undergo SLT, only one eye was used based on which eye had the worse glaucoma as determined by mean deviation on VF assessment), eyes that underwent significant ocular or other glaucoma surgery (including trabeculectomy, OMNI canaloplasty, enucleation, Ahmed, or Baerveldt tube shunts) during the 12-month post-SLT period, patients that were erroneously marked as having received SLT, eyes that lacked significant postoperative data due to loss of patient follow-up. Eyes of patients that underwent YAG capsulotomy during the 12-month postop period after SLT were not excluded.

### 2.3. iStent Surgical Technique

The technique for surgical implantation of first-generation iStent was performed as described elsewhere [19,20,21,22,23]. 

### 2.4. Selective Laser Trabeculoplasty Technique

SLT was performed with the Selecta IITM (Lumenis Inc, San Jose, CA, USA) focused with a Hwang-Latina 5.0 SLT gonio lens onto the pigmented trabecular meshwork. Twenty minutes prior to the procedure, IOP was checked with Tonopen, and patients received a topical miotic (1% pilocarpine) and alpha-2 agonist (brimonidine 0.2%, unless the patient was allergic) in the treated eye. Immediately before the procedure, patients received topical anesthesia to the treatment eye (0.5% proparacaine) and Akten gel on the gonio lens. Laser power started at 0.9 mJ per application and titrated higher until champagne bubbles were visualized upon pulse but not higher than 1.3 mJ per shot. Patients received 90–120 applications along all 360 degrees of the patient’s pigmented TM. In the patients who had SLT post iStent, the SLT was applied to the entire 360 degrees, avoiding the body of the iStent within Schlemm’s canal as well as the tip facing the anterior chamber by 1–2 laser burn widths. Fifteen to thirty minutes following the procedure, IOP was checked by Tonopen for postoperative pressure spikes; patients who had significantly increased postoperative IOP in the treatment eye received an additional drop of brimonidine 0.2%. Patients were then advised to use prednisolone four times daily for three days, to use artificial tears as needed, and to continue their prior glaucoma medications after SLT.

### 2.5. Statistical Analysis

After implementing our exclusion criteria, we ended with data from 128 eyes that underwent primary SLT without a history of iStent and fifteen eyes that underwent SLT after iStent failed to control the patients’ disease progression or failed to control IOP below target. We used the statistical software R to conduct both univariate and multivariate analyses to determine if iStent was statically associated with the outcomes of interest [32]. Specifically, we conducted univariate (unadjusted) and multivariate (adjusted) linear regression modeling, as our outcome variable was continuous for both medication and IOP rather than if there was a reduction or not. We conducted a multivariate analysis to adjust for variables that could have an effect on the association with the outcome variable. We wanted to control for these variables to ensure that this association truly existed [32,33,34]. Our univariate analyses were determined statistically significant at *p* < 0.10. If the iStent was statistically associated with the outcome of interest, we then proceeded to conduct a multivariate analysis. Our multivariate analyses adjusted for age, gender, and either pre-operation number of medications or pre-SLT intraocular pressure depending on the model. Our multivariate analyses were considered statistically significant at *p* < 0.05. A complete analysis was also performed with all covariates included in the model. Although sex and age were not significant, we included them in line with other extensive studies, regardless of significance.

## 3. Results

We observed 143 participants in this analysis. The average age of the participants was 69.91, with the youngest participant being 33 and the oldest 101 years of age, with about 57% of the study population being females. Non-Hispanic Caucasians made up the majority of study participants (69.50%), followed by African American participants (23.40%). The average pre-operation intraocular pressure was 15.93, with the lowest being seven and the highest being 32. Observing the number of medications that patients took before their operation, the average was 2.24, with some patients taking none and the most being five (Table 1 and Table 2).

We conducted independent univariate analyses to determine if the variables age, gender, iStent, race/ethnicity, and initial IOP reading were statistically associated with the outcome of percent IOP reduction post-SLT. The variables were considered statistically significantly associated at *p* < 0.10. We found that both iStent (*p* = 0.082) and initial IOP pre-SLT (*p* < 0.0001) were statistically associated with the outcome of the percent reduction in IOP post-SLT (Table 3).

Next, we conducted a similar analysis for the outcome for the number of medications reduced post-SLT. We conducted independent univariate analyses to determine if the variables age, gender, iStent, race/ethnicity, and the initial number of medications pre-SLT were statistically associated with the outcome. We found that both iStent (*p* < 0.0001) and the initial number of medications pre-SLT (*p* = 0.013) were statistically associated with the number of medications reduced post-SLT (Table 4).

We then conducted a multivariate analysis using the statistically significant variables with the outcome of IOP reduction post-SLT at *p* < 0.10 in the univariate analyses. These variables included iStent (*p* = 0.081) and pre-SLT IOP (*p* < 0.0001). We found that in this multivariate analysis, iStent (*p* = 0.222) was no longer statistically significant with the outcome of IOP reduction post-SLT. The initial IOP pre-SLT (*p* < 0.001) remained statistically significant with the outcome of IOP reduction post-SLT at *p* < 0.05 (Table 5).

Similarly, we conducted a multivariate analysis for the outcome of medication reduction post-SLT using the statistically significant variables with the outcome in the univariate analyses. We included both iStent (*p* < 0.001) and initial number of medications (*p* = 0.013). In this multivariate analysis, we found that both iStent (*p* < 0.0001) and the number of medications pre-operation (*p* < 0.001) were statistically associated with the outcome of medication reduction at *p* < 0.05 (Table 6).

We then completed a multivariate analysis with all of the variables of interest, including, age, gender, race/ethnicity, iStent, and pre-SLT IOP [35,36,37,38,39,40]. We found that in the complete multivariate analysis only the initial IOP pre-operation (*p* < 0.0001) was statistically associated with the outcome of percent IOP reduction post-SLT at *p* < 0.05 (Table 6). We then completed a multivariate analysis with all of the variables of interest, including, age, gender, race/ethnicity, iStent, and pre-SLT IOP. We found that in the complete multivariate analysis only the initial IOP pre-operation (*p* < 0.0001) was statistically associated with the outcome of percent IOP reduction post-SLT at *p* < 0.05 (Table 7).

We then completed a multivariate analysis with all of the variables of interest, including age, gender, race/ethnicity, iStent, and initial IOP pre-operation. We found that in the complete multivariate analysis, the initial number of medications (*p* < 0.0001) and iStent (*p* < 0.0001) was statistically associated with the outcome of the number of medications reduced post-SLT at *p* < 0.05 (Table 8).

## 4. Discussion

With the increasing use of MIGS devices in the glaucoma patient population, it is essential to know whether standard laser techniques such as SLT are still a viable option in the case that the MIGS device fails to control glaucoma progression. While our data analysis showed that iStent was not statistically associated with a reduction in IOP in POAG patients who receive SLT, our data support that history of prior phacoemulsification and iStent implantation is associated with a statistically significant reduction of the number of medications post-SLT compared to control patients. Therefore, SLT is successful at lowering the burden of medications in this patient group by about one medication, on average. This is important as nearly 50% of patients who have POAG are not adherent to taking their medications as prescribed. If there is an intervention that may reduce the number of prescribed medications for POAG management, then clinicians may consider SLT as an effective therapy after iStent implantation for this purpose. However, clinicians should know that the presence of an iStent does not necessarily make subsequent SLT more effective at lowering IOP in POAG patients who have failed iStent. In other words, the presence of an iStent does not increase the success of SLT in lowering IOP compared to standalone SLT.

Our study adds to research into whether SLT is an effective method for controlling IOP in patients who have previously failed a prior glaucoma procedure. SLT efficacy post trabeculectomy, ab interno trabeculectomy, and canaloplasty have been investigated in the literature thus far. Francis et al. conducted a prospective, nonrandomized interventional case series of the success of SLT for uncontrolled open-angle glaucoma in twenty-two eyes of twenty patients [28]. This study found that the cumulative rate of success (defined as IOP reduction greater than three mmHg) of SLT after failed trabeculectomy was 16% at 12 months post laser. Zhang et al. also investigated the efficacy of SLT after failure to maintain target IOP through post-trabeculectomy medication in eighteen eyes of sixteen POAG patients; the authors found that 77.7% of eyes experienced greater than 20% reduction in IOP at 6 and 9 months post laser with no serious complications [29]. Töteberg et al. conducted a retrospective, randomized, interventional case series of fourteen eyes in thirteen patients who underwent SLT after failed combined phacoemulsification cataract extraction and ab interno trabeculectomy, or phaco-trabectome [30]. All patients in this study failed to meet pressure reduction goals of 3 mmHg or 20% from baseline IOP, and the median time to failure was 3.6 ± 8 months. Additionally, the authors found that the number of antiglaucoma medications did not change in the study population. Sluch et al. conducted a retrospective chart review of 19 eyes (17 eyes had POAG) in 17 patients who underwent SLT after canaloplasty tracked up to 24 months after SLT [31]. This study showed a success rate of 16% sustained over 2 years of follow-up; these three patients who responded to SLT were more likely to have never received SLT before canaloplasty. Success in this study was defined as a greater than 20% reduction in IOP or less than 20% decrease in IOP, with no decrease in IOP medications after two to four weeks, increased IOP medications, or need for future IOP-lowering surgery. Our analysis found similar results to those found in these studies regarding the efficacy of SLT after a previously failed glaucoma procedure. Our results did not concur with Töteberg et al. regarding the statistical significance of reducing the number of glaucoma medications in patients who received SLT after a prior glaucoma therapy. Our analysis did concur with all aforementioned studies regarding no statistically significant benefit of SLT in patients who failed a prior glaucoma procedure. As the authors of these studies point out, a sort of selection bias is highly likely to describe that patients who have already failed one form of glaucoma therapy are more likely to fail the second form of glaucoma therapy.

The strengths of this study are that, to our knowledge, this is the first time SLT in post-iStent patients has been examined. To increase the validation in interpreting these results, we excluded patients who had previously received any form of laser trabeculoplasty. Published reports have demonstrated that patients who had already received SLT or ALT were not excluded, leading to potential bias in selecting patients who had already failed a prior SLT or ALT treatment.

Limitations of this study are that data were collected only up to 1 year after SLT, and the analysis was conducted at 6 months post-SLT, whereas some reports on the efficacy of SLT are tracked up to 5 years after therapy [9]. Due to this, our sample size was limited. Though our sample size was limited, it included a diverse population, including racial and ethnic minorities. Even with a small sample size, it is important to include populations that may go understudied within research because, if such populations are ignored, health disparities will only further increase [41]. An additional limitation was observing the data retrospectively in electronic medical record history, which could cause additional bias. Data from 6 months post-SLT were used in our study due to the irregularity and inconsistency of patient follow-up at 1, 3, 6, 9, and 12 months post-SLT; since we wanted to increase our statistical power and provide the most comprehensive snapshot, we used the time when data for our patients was most complete, which was at the 6-month mark.

Our failure to show that iStent confers a statistically significant reduction in IOP in POAG patients undergoing SLT may have been complicated by one of our other success criteria: reducing the number of medications. Johnson et al. did not find that POAG patients who reduce their number of medications have a change in IOP [42]. Furthermore, a meta-analysis of SLT data suggests that SLT is equivalent to some topical medication regimens in terms of IOP reduction [8]. However, it is unknown if a patient who receives SLT only to then be taken off a topical medication may have the same IOP if he stayed on the medication and did not receive SLT. If this were true, our study might have found that iStent was associated with IOP reduction in POAG patients receiving SLT if we had kept all patients in our study on the same number and types of medications as they were before receiving SLT. This, in fact, could be a possible future extension of this study.

Since the introduction of the first-generation iStent, a second-generation model, the iStent inject (Glaukos Corporation) was made available in 2018. However, our data collection on iStent patients preceded the advent of the second-generation iStent, so our analysis only concerns the effect of the first-generation iStent. Multiple studies have shown a significant difference in the IOP-lowering effect of iStent inject versus the first-generation iStent used in our study [43,44]. It may be that a different effect is observed in patients who receive SLT after a failed iStent inject.

Our study adds to research into whether SLT is an effective method for controlling IOP in patients who have previously failed a prior glaucoma procedure. Our study furthers knowledge in the field by considering that the presence of iStent may be associated with a greater reduction of glaucoma medications at 6 months post-SLT. This investigation is a pilot study that will serve as a benchmark for future analyses based on the data collected from these patients. This study design and these data can further be used to address whether the success of SLT is associated with other forms of glaucoma or OHT, medication adherence [45,46], number of chronic medical conditions, specific chronic medical conditions, complicated ocular history, or any of a host of variables collected in this population. This study also opens up further questions to be investigated and answered in this field. For instance, whether SLT is effective therapy after other failed TM bypass devices like Hydrus or other minimally invasive glaucoma surgeries (MIGS) like CyPass, Xen, goniotomy, or OMNI canaloplasty. Further study can also be done regarding the effectiveness of SLT with iStent; similarly to Baser et al. [47], one could investigate whether SLT *before* iStent is effective at reducing the progression of glaucoma or lowering IOP compared to patients who only receive iStent.

## 5. Conclusions

Our study concludes that in POAG patients who receive SLT, a history of iStent implantation during phacoemulsification cataract extraction was not a significant factor in lowering patient IOP in the short-term post-laser period. The data also suggest that IOP reduction with SLT is not significantly synergistic with the first-generation iStent. These results suggest that SLT is not significantly more effective in iStent patients than in patients without iStent. However, our data do support SLT as more effective in medication reduction in iStent patients compared to control patients without iStent. Therefore, although there is not a significant IOP reduction compared to controls, there may be a role of SLT as adjuvant therapy for medication reduction in iStent patients.

## Figures and Tables

**Table 1:** Demographic factors of iStent-SLT patients included.

iStent-SLT Patients	*n* = 15
**Age (years)**	**71.73 (53–88)**
Age at iStent intervention	69.80 (52–87)
**Gender**	–
Male	8 (53.33%)
Female	7 (46.66%)
**Ethnicity**	–
Non-Hispanic Caucasian	14 (93.33%)
African American	1 (6.66%)
Hispanic	0 (0%)
Middle Eastern	0 (0%)
Asian	0 (0%)
Multiple Races	0 (0%)
Unknown/unspecified	0 (0%)
**Eye**	–
OD	4 (26.67%)
OS	11 (73.33%)
**IOP**	–
Pre-SLT	15.94 (7–32)
Post-SLT	13.71 (7–32)
**CCT**	595.87 (508–659)
Baseline MD *	−2.91 (−8.04–−0.31)
**BCVA (LogMAR)**	–
Pre-SLT	0.10 (0.00–0.60)
Post-SLT (6 months)	0.12 (0.00–0.60)
**Number of medications**	–
Pre-SLT/Baseline	1.67 (1–3)
Post-SLT	0.73 (0–3)

* No pre-SLT MD data was found for 2 patients who underwent SLT after iStent. Data for this table value represents the average and range for the other 13 patients who underwent SLT after iStent.

**Table 2:** Demographic factors of primary SLT patient data.

SLT Patients without iStent	*n* = 127
**Age (years)**	**69.73 (33–101)**
**Gender**	–
Male	51 (40.48%)
Female	75 (59.52%)
**Ethnicity**	–
Non-Hispanic Caucasian	82 (64.57%)
African American	33 (25.98%)
Hispanic	1 (0.79%)
Middle Eastern	2 (1.57%)
Asian	1 (0.79%)
Multiple Races	1 (0.79%)
Unknown/Unspecified	7 (5.51%)
**Eye**	–
OD	64 (50.39%)
OS	63 (49.61%)
**IOP**	–
Pre-SLT	15.80 (7–22)
Post-SLT	13.77 (7–32)
**CCT**	556.00 (439–652)
Baseline MD *	−9.22 (−31.87–2.35)
**BCVA (LogMAR)**	–
Pre-SLT	0.27 (0.00–2.30)
Post-SLT (6 months) **	0.27 (0.00–2.30)
**Number of medications**	–
Pre-SLT/Baseline	2.31 (0–5)
Post-SLT	2.15 (0–4)

* No pre-SLT MD data was found for 13 patients who underwent primary SLT without history of iStent. Data for this table value represents the average and range for the other 114 patients who underwent primary SLT without history of iStent. ** No 6 m post-SLT BCVA data available for one patient who underwent primary SLT without iStent. Data for this table value represents the average and range for the other 126 patients who underwent primary SLT without iStent.

**Table 3:** Univariate analysis for association with percent reduction in IOP 6 months post-SLT.

Variable	Estimate	95% CI	*p*-Value
**iStent**	−11.878	−25.268, 1.513	0.082
**Age**	0.050	−0.267, 0.368	0.755
**Male sex**	−0.988	−9.409, 7.434	0.817
**Race**	–	–	–
African American	0.292	−10.299, 9.714	0.954
Other *	5.832	−9.362, 21.027	0.449
**Initial IOP**	−2.786	−3.578, −1.993	<0.0001

* Other includes Middle Eastern, Asian, Hispanic, multiple races, and unknown/unspecified.

**Table 4:** Univariate analysis for association with number of medications 6 months post-SLT.

Variable	Estimate	95% CI	*p*-Value
**iStent**	−0.752	−1.139, −0.366	<0.0001
**Age**	−0.0003	−0.010, 0.10	0.938
**Male sex**	0.018	−0.235, 0.271	0.886
**Race**	–	–	–
African American	0.015	−0.286, 0.316	0.920
Other *	−0.076	−0.533, 0.381	0.744
**Initial number** **of medications**	−0.145	−0.259, −0.032	0.013

* Other includes Middle Eastern, Asian, Hispanic, multiple races, and unknown/unspecified.

**Table 5:** IOP reduction multivariate analysis.

Variable	Estimate	95% CI	*p*-Value
**iStent**	−7.337	−19.062, 4.389	0.218
**Initial IOP**	−2.730	−3.526, −1.934	<0.0001

**Table 6:** Medication reduction multivariate analysis.

Variable	Estimate	95% CI	*p*-Value
**iStent**	−0.876	−1.255, −0.498	<0.0001
**Initial number** **of medications**	−0.192	−0.300, −0.084	<0.001

**Table 7:** IOP reduction complete multivariate.

Variable	Estimate	95% CI	*p*-Value
**iStent**	−5.780	−17.952, 6.391	0.349
**Age**	−0.058	−0.374, 0.257	0.715
**Male sex**	−2.469	−10.323, 5.384	0.535
**Race**	–	–	–
African American	4.101	−5.903, 14.105	0.419
Other *	3.179	−10.973, 17.331	0.658
**Initial IOP**	−2.846	−3.675, −2.017	<0.0001

* Other includes Middle Eastern, Asian, Hispanic, multiple races, and unknown/unspecified.

**Table 8:** Medication reduction complete multivariate.

Variable	Estimate	95% CI	*p*-Value
**iStent**	−0.939	−1.329, −0.549	<0.0001
**Age**	0.004	−0.007, 0.014	0.463
**Male sex**	0.179	−0.076, 0.433	0.167
**Race**	–	–	–
African American	−0.046	−0.361,0.269	0.772
Other *	−0.173	−0.625, 0.278	0.449
**Initial number** **of medications**	−0.216	−0.332, −0.100	<0.0001

* Other includes Middle Eastern, Asian, Hispanic, multiple races, and unknown/unspecified.
